# Origin and evolution of animal multicellularity in the light of phylogenomics and cancer genetics

**DOI:** 10.1007/s12032-022-01740-w

**Published:** 2022-08-16

**Authors:** Florian Jacques, Etienne Baratchart, Kenneth J. Pienta, Emma U. Hammarlund

**Affiliations:** 1 Tissue Development and Evolution (TiDE), Department of Laboratory Medicine, Lund University, Lund, Sweden; 2 Department of Laboratory Medicine, Lund Stem Cell Center, Lund University, Lund, Sweden; 3 The Cancer Ecology Center, Brady Urological Institute, Johns Hopkins School of Medicine, Baltimore, USA

**Keywords:** Evolution, Multicellularity, Tissue, Tumors, Phylogenomics, Genetics

## Abstract

The rise of animals represents a major but enigmatic event in the evolutionary history of life. In recent years, numerous studies have aimed at understanding the genetic basis of this transition. However, genome comparisons of diverse animal and protist lineages suggest that the appearance of gene families that were previously considered animal specific indeed preceded animals. Animals’ unicellular relatives, such as choanoflagellates, ichthyosporeans, and filastereans, demonstrate complex life cycles including transient multicellularity as well as genetic toolkits for temporal cell differentiation, cell-to-cell communication, apoptosis, and cell adhesion. This has warranted further exploration of the genetic basis underlying transitions in cellular organization. An alternative model for the study of transitions in cellular organization is tumors, which exploit physiological programs that characterize both unicellularity and multicellularity. Tumor cells, for example, switch adhesion on and off, up- or downregulate specific cell differentiation states, downregulate apoptosis, and allow cell migration within tissues. Here, we use insights from both the fields of phylogenomics and tumor biology to review the evolutionary history of the regulatory systems of multicellularity and discuss their overlap. We claim that while evolutionary biology has contributed to an increased understanding of cancer, broad investigations into tissue—normal and transformed—can also contribute the framework for exploring animal evolution.

## Introduction

The transition from unicellular to multicellular eukaryotes in the shape of animals (metazoans) was one of the most dramatic events in the evolutionary history of life [1]. Despite many investigations into how this transition happened and its underlying genetic innovations, consensus on the drivers of this transition is still lacking. We here seek additional information from tumor evolution to explore the genetic innovations underlying transitions in cellular organization. Although malignant to its host, tumor evolution demonstrates traits of both a unicellular species and of multicellular cell organization. The study of tumorigenesis can lend insights about transitions between uni- and multicellularity, just as evolutionary concepts have been used to advance insights to cancer.

Multicellularity demonstrates profound coordination and cooperation between cells. Particularly the regulation of cell differentiation, *i.e.*, their function and division of labor, changed from being temporal in single-celled eukaryotes (protists) to being spatiotemporal in animals and plants [1–4]. For these purposes, genes for cell adhesion, cell differentiation, and cell-to-cell communication either pre-existed, were co-opted, or appeared in the genomes of animals [5, 6]. Genome sequencing of animal-related protists and animals such as cnidarians, ctenophores, and sponges have allowed the reconstruction of genetic toolkits and founder genes along their evolution [7–10]. These studies, however, provide a complex picture in which many of the expected specific founder genes appeared before metazoans, like genes, known to regulate cell adhesion, cell-to-cell communication, and cell differentiation [6, 11]. These data suggest a gradual acquisition and complexification of gene families responsible for multicellular development and highlight the need for additional models to understand the genetic basis involved in the regulation of multicellularity.

Multicellular organisms are observed in several eukaryotic lineages, such as charophytes (plants and some green algae), brown algae, red algae, fungi, slime molds, and animals [12]. Out of these, some are simple (with no or little cell differentiation, e.g., some green algae) and several demonstrate transient multicellularity (e.g., slime molds). Only plants and animals demonstrate persistent multicellularity that is complex enough to encompass distinct tissues or organs. To some extent, however, this organization of cells within tissues that defines complex multicellularity is reversible when single cells can transform and start the evolutionary trajectory of the cancer clade [13].

**Fig. 1:**
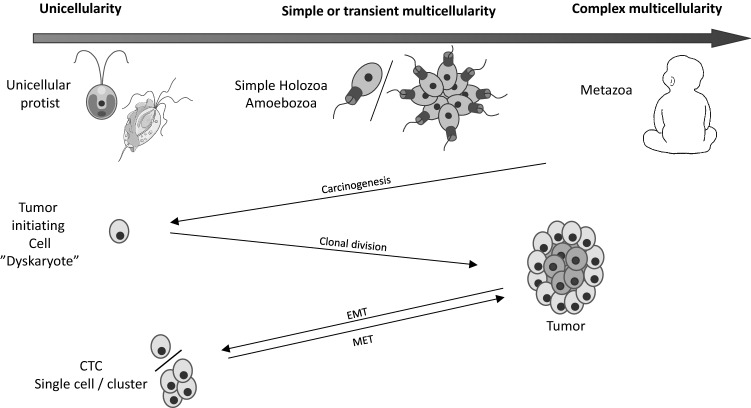
Unicellularity and multicellularity in the evolution of life and of cancer. In both contexts, multicellularity can be transient or permanent and simple or complex. Cancer evolution includes transitions between different states of multicellularity, indicated by black arrows. *EMT* epithelial-to-mesenchymal transition, *MET* Mesenchymal-to-epithelial transition, *CTC* circulating tumor cell

Cancer evolution is thought to be driven by Darwinian selection, where the unit of selection is the single cell. The population dynamics of cancer cells, with sustained proliferation and suppression of cell death, resembles that of unicellular organisms. Cancer cells are also characterized by phenotypic plasticity where cells can de- and trans-differentiate [14]. Hence, cancer progression is sometimes described as an atavistic process (*i.e.*, reappearance of an ancestral character that had been silenced during evolution) and cancer cells as *dyskaryotes* [15, 16] (Box 1). This implies that cancer cells can be fit in their unicellular state and in their multicellular or aggregative configuration (when forming tumors or metastases). For example, the primary tumor can release circulating tumor cells (CTC) that survive on their own through migration via the blood stream, before giving rise to a new round of multicellularity (tumor tissue). This demonstrates collective activities and collaboration with tumor and non-tumor cells [17–21]. The collective activity between cancer cells is further exemplified by how neuroblastoma tumors can exhibit a high degree of cell differentiation, with lobular structures surrounding necrotic cells [22, 23]. Also, angiogenesis within the tumor requires collective cellular activity and collaboration between tumor and stromal cells. Hence, tumor tissue can be regarded as analogous to transient multicellularity that utilize capacities within the acquired toolbox of complex multicellularity (Fig. 1).

Here, we analyze the overlap between genes specific for multicellularity and for cancer. We review the studies from the field of tumor biology that attempts to distinguish the unicellular and multicellular phases of cancer with insights from the fields of phylogenomics and developmental biology. We discuss the role of the overlap in the genetic basis of the transitions between multicellularity and unicellularity during the evolutionary history of animal life and carcinogenesis.

### Box 1 Evolutionary origins of cancer gene categories

Traditionally, cancer drivers can be regarded as affecting basic housekeeping functions within the cell, by mutations of globally expressed genes [24]. These cancer drivers are divided into the three categories: caretakers, gatekeepers, and landscapers [25]. Many cancer drivers, such as TP53, have numerous functions and can be classified in several categories [26].

Caretakers are involved in general cellular processes, such as cell cycle checkpoint, DNA repair, or telomere metabolism and prevent the appearance of mutations [25]. Alterations of caretakers promote carcinogenesis indirectly, by increasing the mutation rate and genome instability. Other mechanisms, like whole-genome doubling and karyotype remodeling, increase the genomic and chromosomal instability of cancer cells and, by doing so, increase population diversity.

Gatekeepers are involved in processes, such as cell growth, proliferation, signaling, death, division, and differentiation. They include proto-oncogenes and tumor suppressor genes [25]. Disruptions of gatekeepers promote tumor progression directly, by altering cell growth, death, and differentiation [14]. Within gatekeepers, tumor suppressors prevent cells from uncontrolled proliferating and proto-oncogenes influence cooperation among cells. For example, mutations of a tumor suppressor like TP53 leads to the removal of controlled cell death (apoptosis) and can together with another mutation be inferred to induce cancer. In contrast, mutations to a proto-oncogene like KRAS can promote uncontrolled cell proliferation and is observed to associate with the aggressiveness of metastasis. For our purposes here, tumor suppressors can also be regarded as ‘multicellularity regulators’ that need to be lost for the reversal to a unicellular state.

Mutations on landscapers affect the stromal environment that can indirectly contribute to neoplastic transformation of the cells [25]. Phylostratigraphic analyses identified two major peaks of appearance of cancer drivers [27]. The first one, corresponding to caretakers, dates to the origin of the first cellular organisms. The second one, corresponding to gatekeepers, dates to the origin of animals. The latter emerged at the origin of multicellularity and ensured collective fitness by enabling collaboration between cells. Domazet-Lošo and Tautz [27] suggest that the evolutionary succession of cancer gene appearance mirrors the ontogenetic succession of cancer progression, where mutations in caretakers precede mutations in gatekeepers.

## Models for the rise of multicellularity

The rise of multicellularity is currently studied within the fields of animal evolutionary history and developmental biology. We describe the current models for the evolution of multicellularity and specifically animals, describing the genetic toolkits that are potentially involved. In parallel, models from the field of tumor biology pertaining to the rise of transformed multicellularity and the genetic toolkits believed to be involved are depicted.

### Leading to animals

In living organisms, transient and persistent multicellularity have arisen several times and through different mechanisms. The two most common mechanisms are clonal division without physical separations of the daughter cells, as in animals, and cell aggregation, as in slime molds [28]. Multicellularity through cell aggregation is less stable since the cells do not share the same genetic material, which leads to intra-organismal competition [29]. Other mechanisms for multicellularity include cellularization of a large multinucleated cell as in fungi [30] or incomplete cell division as seen in cyanobacteria or algae [31].

Animals belong to a group of eukaryotes termed Opisthokonta, which also includes fungi and several clades of protists (unicellular eukaryotes). Studies of the biology of unicellular Opisthokonta have provided valuable information to the transition between unicellularity and multicellularity at the origin of animals. Animals (Metazoa) together with their three groups of unicellular sister taxons of metazoans (choanoflagellates, filastereans, and ichthyosporeans) constitute the holozoans. The holozoan ancestors of animals likely diverged some 0.9 billion years ago (Ga), and with the first complex multicellular animals evolving from unicellular or colonial organisms at some 0.8–0.7 Ga [32]. Holozoans have complex life cycles including different types of transient clonal, aggregative, or coenocytic (multinucleated as a result of nuclear divisions without cytokinesis) multicellularity as well as temporally regulated cell differentiation (Box 2). The advent of complex multicellularity is thought to result from either a division of labor from multifunctional cells or the conversion of unicellular organisms with cell differentiation into connected differentiated cells [33].

Several models have been proposed to explain the origin of animals in the past decades, with insights from embryology, comparative genomics, and transcriptomics. Early on, the models suggested that multicellularity formed first and then cells differentiated within it. For example, Haeckel’s *Gastraea* model [34], was based on his belief that embryonic development recapitulates evolution, stated that the first step of animal evolution would be a gathering of identical cells forming a colony. Then, based on the resemblance between choanoflagellates and the choanocytes of sponges, animals were thought to derive from a colony of cells similar to choanoflagellates [35]. Animals are also proposed to have evolved via juvenile characters (by paedomorphism) from an organism that resembled a planula, *i.e.*, a cnidarian larva [36]. Others suggested that animals derived from an amorphous collection of cells with a gradually developing internal cavity that gave birth to primitive sponge like or a cnidaria-like animal [37]. More recent models take into account that unicellular holozoans already demonstrate cell differentiation. In these models, the ancestor of animals is not considered sponge like, but a collection of convertible cells, capable of transition between multiple states, like archeocytes of sponges and stem cells of modern animals [4, 38]. In the near future, it appears that mechanistic studies of multiple animals should be able to elucidate what modifications to cell interactions and cell-fate regulation that preceded the transition to multicellularity. As of yet, however, we are unable to tease apart to what degree ‘cells getting together’ or ‘cells getting specialized together’ dictated the transition.

### Box 2 Multicellularity in animal-related protist lineages

Several groups of holozoans (i.e., protists related to animals) display transient multicellularity during their life cycle. For instance, choanoflagellates form colonies by clonal division under favorable conditions [39, 40]. Filastereans and ichthyosporeans, also known as mesomycetozoans, are parasites or commensals of animals numbering only a few described species [41–44]. The filasterean life cycle includes an amoeboid stage, a cystic stage, and an aggregated multicellular stage forming under starvation [45]. Ichthyosporeans on the other hand begin with a mononucleated cell that transforms into a multinucleated coenocyte and releases mononucleated cells after cellularization [46–48]. The life cycle of slime molds, another group of protists related to animals and fungi [49], also comprises a succession of unicellular and multicellular phases, the latter associated with sexual reproduction. Under adverse conditions, such as starvation, they form a multicellular aggregate that differentiates into a stalk and a fruiting body containing encapsulated dormant spores [50]. Under favorable conditions they germinate into amoeboid biflagellate haploid cells that combine with each other into a diploid zygotic slime mold [50].

### Leading to metastatic tumors

Cancers derived from epithelial tissues (carcinomas) are the most common type of cancer. During their evolution, carcinoma cells exhibit plasticity between epithelial and mesenchymal phenotypes, endowing them with invasive and migratory properties. These cancer cells can eventually spread into the extracellular matrix, lymphatic, and vascular systems to start a metastatic cascade and colonize distant organs. At the new sites, new tumor tissue (metastases) is ultimately lethal to the host organism [15, 51]. The cause behind the first cancer cell remains debated, with discussions of, e.g., key mutations, a decline in the tissue homeostasis and epigenetic alterations [52–56]. Here, we focus on how cancer cells from the primary tumor can pass through a unicellular phase that subsequently seeds new units of transformed tissue.

The unicellular cancer cell is characterized by competitive growth and replicative immortality [53, 57]. The single cancer cell can also survive on its own by suppressing cell-to-cell communication mechanisms responsible for apoptosis. This allows the cancer cell to escape cell death that would meet other normal somatic cells, when the cell is no longer in contact with the extracellular matrix, called *anoikis* [58]. The epithelial-to-mesenchymal transition (EMT) also favors increase plasticity of the cell as in unicellular organisms [53]. The formation of metastases, however, requires to some extent a reversal toward more differentiated cell states again. Cancer cells that have undergone EMT must again go through the mesenchymal-to-epithelial transition (MET) and form a multicellular tissue [53]. Besides the ability to form somewhat organized and differentiated tissues, the so-called cooperation theory proposes that tumor cells could be able to communicate through the sharing of molecules and develop cooperative defense strategy against the immune system [59]. Hence, tumors can be interpreted as a new form of multicellularity resulting from de- and re-differentiation mechanisms.

To our knowledge, only a handful of studies have explored genetic alterations in cancer cells demonstrating a switch between unicellularity and multicellularity during cancer progression. These studies suggest that ancient genes related to multicellularity are under positive selection or hypermutated during tumor evolution, while some oncogenes, in contrast, represent unicellular processes. For example, many genes dysregulated or under positive selection in cancer progression toward metastatic tumors are related to multicellular development. These genes are involved in cell-to-cell adhesion, such as integrin, cadherin, catenin, and TGF-β, and date back to the origin of animals [21, 60]. That ancient genes (also found in protists and bacteria) also hypermutated and overexpressed in cancer were demonstrated by [61]. Also, [62] noted an upregulation of ancient genes in normal animal polyploid tissues. This led to the proposition that the phylostratigraphic shift to unicellular-like organisms or stem-like cells could be partly associated with polyploidy, which is frequent in tumors [63]. Cancer cells have also been characterized to lose their ability to regulate unicellular processes through dozens of driver mutations [57, 60]. These cancer drivers are critical for the control of unicellular processes in a multicellular context and are suggested to provide points of vulnerability in the frame of cancer [60], [57]. Hence, it can be argued that cancer results from an alteration of the control of unicellular machinery by multicellularity-related pathways and a phylostratigraphic shift toward a unicellular-like state. Therefore, multicellularity-related pathways, such as cell adhesion and cell communication, are critical for cancer development and represent an appealing target to better understand the multistep nature of cancer evolution.

## The genetic toolkits of multicellularity in animal and cancer evolution

Genetic underpinnings to the rise of multicellularity have been explored for decades. The fields of geobiology, developmental biology, and tumor biology, in parallel, have sought specific genes that could explain the transitions between uni- and multicellularity. We briefly present examples of the evolution of genetic toolkits within Holozoan organisms, and how these are known to also play roles during tumor evolution.

Solutions for cell-to-cell communication, cell adhesion, and cell differentiation are believed to be regulated by both de novo appearance and co-option of genes [64]. Since a large repertoire of genes for multicellularity appear to be present in the genomes of unicellular holozoans, the acquisition of multicellularity appears accompanied particularly by a co-option and expansion of many genes related to these functions [64]. In addition, the evolution of transcription factors’ families appears important for the acquisition of multicellularity. For example, cell differentiation and organogenesis are highly dependent on transcription factors that regulate spatial expression of genes and cell-fate specification. Below follows a presentation of some of the primitive regulatory capacities of cell adhesion and cell differentiation that existed before multicellularity and were later co-opted to form distinct tissues of different cell types in animals. Within tissues, furthermore, the alterations of systems that are at the core of multicellularity, including cell adhesion, cell-to-cell communication, and the regulatory genome, are characteristic of carcinogenesis.

### Cell–cell and cell–extracellular matrix adhesion

**Fig. 2:**
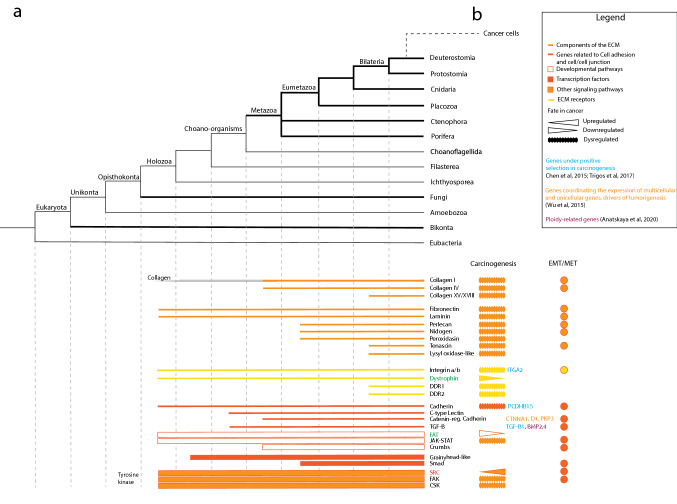
Cladogram representing the origin and expansion of the genetic toolkit for cell-to-cell adhesion and elaboration of extracellular matrix in animals (**a**). Bars represent the presence of the corresponding protein or protein family based on genome comparisons. The fate of these toolkits in cancer is also marked if upregulated, downregulated, or dysregulated, and families that include genes involved in EMT/MET are indicated (**b**). Proto-oncogenes are marked in red; tumor suppressors are marked in green (Color figure online)

Unicellular holozoans possess many genes encoding protein involved in cell-to-cell and cell-to-ECM adhesion proteins (Fig. 2). In the genome of choanoflagellates, more than 20 families of predicted cadherin are identified [65, 66]. In a filasterean, a complete integrin adhesome is observed, including Integrin α and β, tyrosine kinases, and all other components of animal integrin adhesion complex. That this integrin adhesome is absent in choanoflagellates is supposed to result from secondary loss [6]. Also, unicellular holozoans demonstrate components of the extracellular matrix that are key in animals, such as laminin, dystrophin, collagen, and fibronectin. Some of these are shared by animals and choanoflagellates, such as C-type lectin, a protein of the extracellular matrix that binds carbohydrate in a calcium-dependent manner [65]. Other proteins appear restricted to animals, such as TGF-β and catenin, a family of proteins involved in cell adhesion by linking cadherins to the actin filaments of the cytoskeleton [19, 66]. With eumetazoans (bilaterians and cnidarians), other components of the extracellular matrix appeared, such as perlecan, nidogen, and peroxidasin (Fig. 2). Bilaterians uniquely possess tenascin (a glycoprotein composing the extracellular matrix), lysyl oxidase (involved in collagen stabilization), and discoidin domain receptors (receptor tyrosine kinases that bind collagen) [11]. Hence, many proteins mediating cell–cell adhesion were present before the appearance of permanent multicellularity, but a more complete adhesion system appeared with animals and underwent further complexification along the evolution of bilaterians.

In tumor evolution, cell adhesion is noted to be altered, and some genes associated to it appear under positive selection and maybe associated to the processes that are altered in uni- and multicellularity. For example, integrins and other components of extracellular matrix, such as fibronectin, lectin, and laminin, are aberrantly produced in cancer and involved in metastasis [67]. Collagens I, III, and IV are abnormally expressed in some cancers which lead to, e.g., chemotherapy resistance (Fig. 2) [68]. Furthermore, collagen XV has been proposed to be a tumor suppressor [69]. Also, several genes involved in cell adhesion (e.g., as members of the integrin, cadherin, and TGF-β families) were noted to be under positive selection during carcinogenesis, in a study of experimental evolution on xenograft tumor [21]. Trigos et al. [57] emphasize that adhesion genes bridge unicellular and multicellular processes by how it regulates cadherin, catenin, and integrin binding, as well as cytoskeleton assembly [57]. More specifically, the cell–cell adhesion and cell–ECM adhesion systems play key roles in EMT and MET transitions (Fig. 2). These systems are co-opted by cancer cells to invade mesenchymal tissues and colonize new organs. During EMT, the colonization of the ECM is achieved by downregulating mechanisms of cell–cell interaction, such as E-cadherin, and upregulating mesenchymal markers, such as N-cadherin, fibronectin, and β1 and β3 integrins. Activation of EMT/MET is coupled to components of the extracellular matrix, such as collagen I and IV, fibronectin, laminin, nidogen, peroxidasin, and integrins (for review, see [70]). It appears fair to conclude that cancer cell migration and metastasis strongly rely on switching on and off cell-to-cell and cell-to-ECM adhesion systems.

### Cell-to-cell communication pathways

**Fig. 3:**
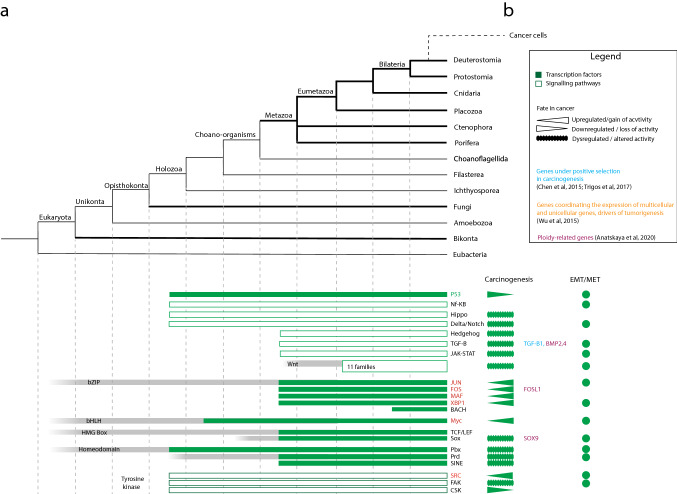
Cladogram representing the evolutionary origin and expansion of the genetic toolkit for cell communication in animals (**a**). Bars represent the presence of the corresponding protein or protein family based on genome comparisons. The fate of these toolkits in cancer is also marked, if upregulated, downregulated, or dysregulated, and families that include genes involved in EMT/MET are indicated (**b**). Proto-oncogenes are marked in red; tumor suppressors are marked in green (Color figure online)

Unicellular holozoans possess signal transduction systems and cell-to-cell communication systems that are key to multicellular development (Fig. 3). Genes that are key for the regulation of embryogenesis and cell differentiation in animals (e.g., TP53, a major regulator of cell cycle and apoptosis, and Delta/Notch as well as a hedgehog-related gene) have been identified in the genome of choanoflagellates [64, 71–74]. Another pathway that regulates cell proliferation and apoptosis in animals, the Hippo signaling pathway, has also been identified in a filasterean [75]. Proper development is also regulated by tyrosine kinases that respond to extracellular growth factors and mediate signaling between cells. Some cytoplasmic tyrosine kinases (e.g., SRC, FAK, and CSK are also present in unicellular holozoans [48, 65, 76], where they regulate cell proliferation in response to environmental conditions, such as nutrient availability [76]. Hence, many features of animal cell communication pathways were already present in their unicellular ancestors. Animals uniquely demonstrate some signaling pathways, such as JAK-STAT, Wnt, and TGF-β [72] (Fig. 3). The Hippo signaling pathway also complexified, with the appearance of the upstream receptors Fat and Crumbs [12]. Hedgehog and components of the Notch pathways are thought to thereafter have been secondarily lost in placozoans and ctenophores [64].

During carcinogenesis, mutations related to cell cycle control, apoptosis, and genome integrity are under strong Darwinian selection [61]. Moreover, signaling pathways related to cell-to-cell communication are mutated, overexpressed, attributed to be tumor suppressor or oncogenes, and associated with transitions in cell phenotype (Fig. 3. In many cancers, the pathways for cell-to-cell communication (e.g., NF-κB, Delta/Notch, and JAK-STAT are dysregulated [77]. Mutation can activate Hedgehog signaling, which modulates tumor growth [78]. Many genes of the Hippo signaling pathway, involved in the control of cell proliferation and apoptosis, are even described as tumor suppressors or oncogenes [79]. Tyrosine kinases, involved in cell differentiation, metabolism, adhesion, and cell death, are dysregulated in cancer. Some, such as Src, are proto-oncogenes [80]. For the context here, the loss of a tumor suppressor gene that leads to uncontrolled tissue formation may represent the loss of the regulation of functional multicellularity. The transition of cancer cell phenotype is also associated to cell-to-cell communication pathways. For example, TGF-β, Notch, Wnt, NF-κB, and tyrosine kinases are involved in EMT/MET mediation [70]. Genes involved in both maintenance of stemness in stem cells and in cell differentiation can be overexpressed or dysregulated in cancer [80–82]. In the case of circulating tumor cells (CTCs), specifically, these signaling pathways (e.g., Wnt) are highly expressed and involved in *anoikis* suppression [83, 84]. Hence, cancer progression is highly dependent on the deregulation of cell-to-cell communication pathways mediating cell proliferation, cell differentiation, cell death, and control of cell stemness.

### Regulatory genome and cell differentiation and de-differentiation

**Fig. 4:**
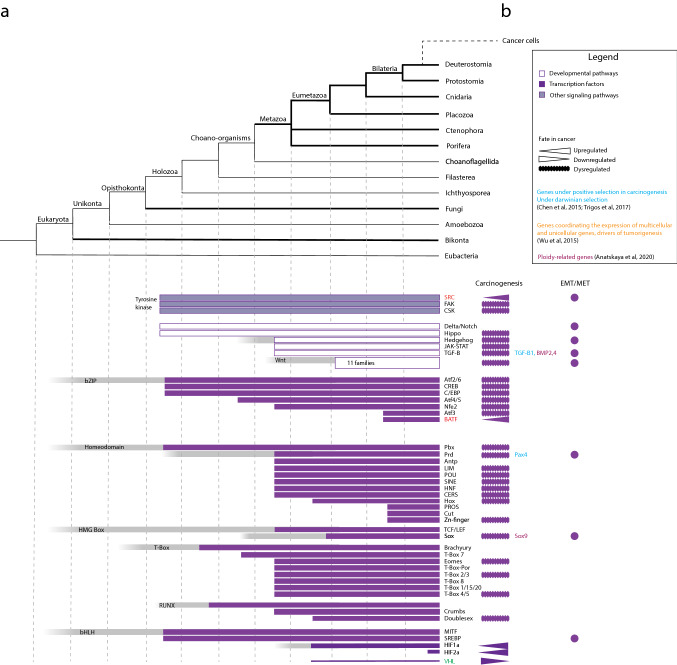
Cladogram representing the evolution of the genetic toolkit for cell differentiation, organogenesis, and multicellular development in animals (**a**). Bars represent the presence of the corresponding protein or protein family based on genome comparisons. The fate of these toolkits in cancer is also marked, as upregulated, downregulated, or dysregulated, and families that include genes involved in EMT/MET are indicated (**b**). Proto-oncogenes (red) and tumor suppressors (green) are marked (Color figure online)

Animal tissues and cell phenotypes are generated with gene regulatory networks that drive specific families of transcription factor. For example, cell differentiation and organogenesis are regulated through transcription factors (TF), such as homeobox, T-box, bHLH, RUNX, and bZIP. These TFs are identified in the genomes of unicellular holozoans, although some of them have been secondarily lost in choanoflagellates [64]. For example, a filasterean demonstrates orthologues of cell proliferation and motility regulators, such as the bHLH Myc and the T-Box Brachyury [64, 73]. This suggests that these transcription factors enabled early forms of cell differentiation that preceded multicellularity but was further expanded upon at the origin of animals [64] (Fig. 4).

Animal-specific TFs are members of the Homeodomain class that regulate cell differentiation, embryonic development, and organogenesis (e.g., Atnp, Prd, and Hox) and members of T-box, bHLH, zinc-finger, and HMGBox superfamilies, such as Sox [64, 72]. Also classes of transcription factors are novel, for example, Ets, Smad, Irf, nuclear receptor, and doublesex appeared with animals [64]. Other regulatory capacities that appeared at the origin of metazoans were distal enhancers promotors types I (adult) and promotors type III (developmentally regulated) [32, 85]. Overall, this expansion of the gene regulatory systems can have allowed an increased complexity of spatiotemporal cell differentiation and, thus, an increased capacity for tissue morphologies [64]. After the origin of metazoans, this expansion of transcription factors and signaling pathways continued and involved in cell differentiation and multicellular development. With Eumetazoans, the Wnt superfamily diverged into 11 families in Eumetazoans with its last subfamily appearing in vertebrates [86]. This expansion appears important since Wnt signaling plays a key role in the control of body architecture, cell differentiation, and cell proliferation control during embryogenesis. Also, the first Hox gene, involved in the control of axial patterning during early development, appeared with Eumetazoans (for review, see [87]). The Hox family further expanded at the origin of bilaterians, and their specific cluster organization appeared in vertebrates, which testaments for its role for a greater complexity of animal morphology and embryogenesis.

An aspect of animal tissue maintenance that remains less emphasized in discussions pertaining to the animal origins is that of cell-fate alterations [88]. Development and tissue homeostasis are highly dependent on cells to not only differentiate but also to alter their fate by, e.g., de-differentiation. Another representation of the capacity to alter cell fates would be maintaining the immature cell phenotype (e.g., stem cells) at conditions that otherwise drives differentiation, such as oxic conditions. Stem cells are characterized by their ability of self-renewal and the breeding of different cell types. In mammals, the stem cell phenotype is known to be promoted by hypoxic conditions [< 1–3% O_2_ and oxygen-sensing mechanisms (for review, see [89]). The main known actors of the oxygen-sensing system are transcription factors of the bHLH family called Hypoxia-inducible factors HIFs, which induce the transcription of specific genes involved in stem cell maintenance. In oxic conditions, HIFs are targeted by the von Hippel–Lindau protein (pVHL for their ubiquitylation and proteasomal degradation. HIF-1α and pVHL are present in all eumetazoans, while a second member, HIF-2α, appeared in vertebrates [90–93] (Fig. 4). The acquisition of HIF-α by eumetazoans is suggested to have allowed animals to dwell in environments with fluctuating oxygen conditions and, for particularly vertebrates, to improve regulation of cell stemness and cell de-differentiation [94]. Hence, cell differentiation pathways were already present in unicellular holozoans but their expansion appears to have played major roles over the evolution of animals for both their capacity to regulate cell-fate regulation (forward and backward along the differentiation spectrum), alteration, and maintenance.

In cancer, transcription factors involved in cell differentiation are often up- or deregulated (Fig. 4). For example, the T-Box, RUNX, and Homeobox families contain both tumor suppressors and tumor promotors [95, 96]. Several members of the bHLH superfamily regulate cell fate, like the well-studied Myc, are proto-oncogenes. Homeobox genes that are normally expressed in undifferentiated cells are upregulated in cancer, while those expressed in differentiated tissues are downregulated. They are described as “tumor modulators” rather than oncogenes or tumor suppressors [97]. More specifically, transcriptions factors, like Smad and members of the Homeobox and bHLH families, such as Prd and Twist, are directly involved in switching cells from the epithelial state (EMT) to a mesenchymal state (MET) [70]. Differentiated tumor cells arise from the division and specialization of cancer cells with stem cell-like properties, including self-renewal, de-differentiation, and proliferation, called cancer stem cells. Stemness of cancer cells associates to mutations and epigenetic changes affecting cell differentiation and embryogenesis pathways, such as Wnt, β-catenin, Hedgehog, and Notch. These pathways are also involved in the re-differentiation of the cells resulting from the division of cancer stem cells [98]. HIF-1α and HIF-2α can promote de-differentiation and a stem-like phenotype of cancer stem cells that are critical for EMT/MET transitions, even in non-hypoxic conditions [99, 100] (Fig. 4). Tumor development due to the inactivation of the pVHL tumor suppressor or gain of function of HIF-2a, both leading to an activity of HIFs at physoxic conditions, has been coupled to several forms of cancer [101–103]. The implications of the disruption of oxygen-sensing mechanisms, which are at the core of cancer evolution, highlight the importance of controlling trans- and de-differentiation for the success of tumor multicellularity.

## Discussion

The maps presented here depict the view described also by others in the last decade—there is not one specific increase of gene diversity or regulatory networks associated with the appearance of Metazoa. In contrast, genetic diversity and regulatory networks expand in a stepwise manner from the divergence of Opisthokonta to vertebrate animals. Below, we discuss this expansion of gene diversity and regulatory networks over animal history, its overlap to tumor multicellularity, and how the importance of alteration of cells fates may remain under-appreciated in discussion of multicellular evolution.

Our maps demonstrate that the transition from simple multicellularity to persistent multicellular development is associated with a significant complexification, particularly of the regulatory genome and the cell-to-cell communication systems. On one hand, animal multicellularity utilizes regulatory capacity through cell communication pathways and transcription factors that protists lack, such as Wnt, and many Homeodomain and bZIP transcriptions factors. On the other hand, toolkits for extracellular matrix, cell–cell, and cell–extracellular matrix adhesion expanded drastically at the divergence of Holozoa, Metazoa, and Bilateria. These toolkits associate with a more elaborate tissue organization [66]. For example, transcription factor families (e.g., Homeobox and T-box) and signaling pathways known to regulate early development and morphogenesis [104]. A genetic expansion appears also at the divergence between invertebrates and vertebrates. For example, vertebrates demonstrate refined capacities for oxygen sensing and the maintenance of cell stemness in specific tissue niches [91]. Overall, the expansion testifies to how the capacity to form complex tissues is unraveled by and within bilaterians [105, 106].

An overlap between genes involved in animal and cancer evolution has become clear over recent years. Our maps demonstrate how many of the same genes and pathways identified as specific to animal multicellularity are exploited, dysregulated, or selected for during carcinogenesis (Figs. 2, 3, 4). Among these are transcription factors (e.g., Homeodomain, T-Box and bHLH), cell differentiation pathways, cell adhesion systems (*e.g.*, cadherins, integrins, and collagen), oxygen-sensing mechanisms, and cell communication pathways (*e.g.*, TGF-β and tyrosine kinases). Notably, these genes are involved in transitions between unicellular-like and multicellular-like entities. For example, EMT (when cells are motile and mesenchymal) represents a loss of interaction with the other cells and a gain of interaction with components of the extracellular matrix. In contrast, MET (when cells get polarized and epithelial again) is linked with increased interaction and the generation of new multicellularity. It appears that these genes are particularly important for the switching between phenotypic modes within tumor evolution [57, 60].

Phenotypic plasticity to switch between uni- and multicellularity is widespread in eukaryotic clades, including some green algae, fungi, and choanoflagellates [107, 108]. It appears that the regulatory genome of unicellular ancestors of animals was sufficient to ensure a primitive form of cell differentiation and transient multicellular development,a capacity that was inherited by animals [64]. However, while the emerging picture indicate that cells were multifunctional before and at the dawn of Metazoa, modifications of these functions occurred throughout animal evolution [88]. One important such modification would be how cell identities can be altered. It has become increasingly clear that cell- fate alteration is important during tissue formation, maintenance, and transformation.

Compared to the need of cell specialization, it is much less discussed that the alteration of cell identity also plays a fundamental part in tissue function and animal development [109]. This is a new field of investigation driven by medical in vitro work to reinvigorate the human regenerative capacity. A dramatic example of its advancement occurred in 2006 when specialized cells were induced to become pluripotent stem cells (iPSC), as demonstrated by Takahashi and Yamanaka [110]. However, trans- and de-differentiation are necessary also for normal tissue homeostasis. Cellular trans- and de-differentiation are described from e.g., Hydra, Planaria, and newts [111–113]. Mammalian tissues have a more limited capacity to regenerate than for example newt, but it has become more apparent that physiological stresses can lead to changes in cell identities [109]. Also, cell-fate commitment during in normal tissue and development is described to be tightly regulated by, e.g., the vertebrate-specific HIF-2a [114]. This would imply that the most complex animals (organ-grade tissues with the highest number of cell types) are also those that granted the most versatile mechanisms for cell identity alterations [91, 94]. The long lifespan of vertebrates is also that most severely affected metastatic cancers. Thus, the phenomena to alter cell identity appear conserved within the animal kingdom and may be involved in carcinogenesis within particularly in vertebrates.

Tumor evolution critically utilizes plasticity in cell phenotypes and its features of both uni- and multicellularity. When cancer cells appear in mammalian tissues, the capacity to switch back to unicellularity and between cell fates are reborn or enhanced. Indeed, cancer is described as a speciation event within the organism and requires at least one reversal to the single cell to survive on its own [13]. Within subsequent tumor evolution also, genes involved to switch fates between, e.g., epithelial and mesenchymal cell types are involved in cancers (see Figs. 2, 3, 4). Arnatskaya and co-workers take it even further and claim that common to cancers is the mere capacity to switch cell between uni- and multicellularity [62]. Indeed, unlocking cell plasticity has been suggested as a hallmark of cancer [14], which allows cancer cells to switch behaviors, like uni/multicellular, depending on the context, making them highly adaptable. This suggests a fitness advantage of cells able to switch between unicellularity and multicellularity, which parallels the case of organisms that display transient multicellularity, such as some protists, fungi, or algae. This would mean that while we advanced insights to what allows for multicellularity to form over the evolution of animals, tumor evolution uses tools also for its reversal. At the core of this reversal, we claim, lies the capacity to alter cell fates. If the capacity for cell-fate alterations increased over animal evolution, cancer of vertebrate animals would possess the utmost options for cell and tissue plasticity at hand.

Parallel to the role of genes, however, novel insight from both ecology and tumor biology highlights that diversification is driven by other and complex interactions. For example, eco-evolutionary principles demonstrate how different environmental niches select for different phenotypes, such as uni- or multicellular units. When different phenotypes coexist within a tumor, the Darwinian units of selection are the single cells [115–117]. These cells increase their fitness by accumulation of genetic and non-genetic alterations that provide them with a selective advantage. In addition to competition, many phenotypes observed in cancer also rely on cooperation, making the concept of group phenotype as relevant as cellular phenotype [18–21, 59, 63]. For example, when new blood vessels within the tumor form through production of pro-angiogenic factors from existing vessels such a phenotype requires cooperation of multiple cell types, including cancer cells, endothelial cells, and stromal cells. Other examples of complex multicellular behavior in cancer have been observed across studies. For instance, certain tumor phenotypes increase the fitness of their neighbors by production of pro-growth factors [118], a so-called non-cell autonomous mechanism. Hence, like in the evolutionary history of animals, the eco-evolutionary transition from competition to cooperation, where the unit of selection shifts from the cell to the phenotypic group [118], is likely crucial for the transition from unicellularity to multicellularity in cancer evolution.

To summarize, the genetic underpinnings to the rise of animals on Earth are far from straight forward. Still, an overlap between animal and cancer-related genes and pathways is curious. While this overlap can be seen to reflect how tumor evolution masters transitions between uni- and multicellularity [21, 24, 52], we emphasize that its mastery hinges regulatory capacities for both the establishment and reversal of cell fates and tissue integrity. Although eco-evolutionary dynamics play a large part in what genotype becomes successful, broad studies into both what allows the establishment and reversal of multicellularity may be beneficial for the fields of tumor and animal history.

## Data Availability

Not applicable.
